# Supplementary material for: An integrated enzymatic and computational pipeline for quantifying off-target base-editing
Source: bioRxiv. 2025 Aug 26:2025.08.26.667396. Preprint. [Version 1] doi: 10.1101/2025.08.26.667396 (PMC12407836; doi:10.1101/2025.08.26.667396)
Supplement: Supplement 3 — Supplementary Table 4. Donors used in the study and marked for usage in iGUIDE/BEiGUIDE and/or rhAmpSeq experiments. [file media-3.docx]

**Supplementary Table 4. Summary of donor samples used in the different experiments**

| **Donor** | **iGUIDE/BEiGUIDE** | **rhAmp-Seq** |
| --- | --- | --- |
| pND579 | 1 | NA |
| pND567 | 1 | NA |
| pTMP491 | 1 | NA |
| p08718-039 | 1 | NA |
| p08718-032 | 1 | 1 |
| p08718-043 | 1 | NA |
| pND608 | 1 | 1 |
| pND658 | 1 | NA |
| pND607 | 1 | NA |
| pND578 | NA | 1 |
| pND500 | NA | 1 |
| pND627 | NA | 1 |
