# Supplementary material for: An integrated enzymatic and computational pipeline for quantifying off-target base-editing
Source: bioRxiv. 2025 Aug 26:2025.08.26.667396. Preprint. [Version 1] doi: 10.1101/2025.08.26.667396 (PMC12407836; doi:10.1101/2025.08.26.667396)
Supplement: Supplement 6 — Supplementary Table 7. The number of predicted off-target sites for all cut-predicting bioinformatic tools. Both the total number of dsODN incorporations and in transcription unit (in_TU) are listed. [file media-6.docx]

**Supplementary Table 7. Summary of predicted off-target sites for BE8 gRNA Cas9 cleavage using 10 different in silico tools**

| **method** | **total_hits** | **in_TU** |
| --- | --- | --- |
| ABEdeepoff | 745 | 308 |
| CasOFFinder | 745 | 308 |
| CCTop | 322 | 117 |
| CHOPCHOP | 44 | 18 |
| COSMID | 11 | 6 |
| CRISPOR | 808 | 259 |
| CRISPRme | 968 | 384 |
| CRISPRoff | 50 | 6 |
| CRISTA | 3181 | 1234 |
| IDT | 96 | 36 |
