# Supplementary material for: An integrated enzymatic and computational pipeline for quantifying off-target base-editing
Source: bioRxiv. 2025 Aug 26:2025.08.26.667396. Preprint. [Version 1] doi: 10.1101/2025.08.26.667396 (PMC12407836; doi:10.1101/2025.08.26.667396)
Supplement: 1 [file NIHPP2025.08.26.667396V1-supplement-1.pdf]

bioRxiv preprint doi: https://doi.org/10.1101/2025.08.26.667396; this version posted August 26, 2025. The copyright holder for this preprint (which was not certified by peer review) is the author/funder, who has granted bioRxiv a license to display the preprint in perpetuity. It is made available under aCC-BY 4.0 International license.

Supplementary Figures

Supplementary Figure 1. Comparison of marking efficiency over three cell donors (indicated by the three colors and key at right). The x-axis shows the enzyme used, the y-axis shows the efficiency of marking (% of oligonucleotide incorporation at *PTPRC* as a % of the total).

Supplementary Figure 2. Comparison of off-target sites identified in the iGUIDE data and using bioinformatic methods. A) Principal coordinate analysis to compare the aggregate rankings of sites by each method. Both marking by oligonucleotide incorporation (iGUIDE) and bioinformatic methods are shown. The methods used are indicated by the color code to the right. The percent variance explained by each axis is shown in parenthesis. B) The within-group rank similarity of the top ten targets of *PTPRC* sgRNA, with method listed on the x-axis. Distribution is composed of biological replicates. Similarity was assessed using Kendall's Tau. C) Between-group rank similarities, with pair-wise comparison of biological replicates in method group (x-axis) to biological replicates from method groups displayed in the legend.

Supplementary Figure 3. Comparison of methods for predicting sites of editing compared to results from actual ABE8e editing analyzed using the targeted amplicon panel. Results are compared by quantification of precision (true positives/true+false positives) and recall (true positives/true positives+false negatives) for the target sites analyzed by each method and compared to "truth"

bioRxiv preprint doi: https://doi.org/10.1101/2025.08.26.667396; this version posted August 26, 2025. The copyright holder for this preprint (which was not certified by peer review) is the author/funder, who has granted bioRxiv a license to display the preprint in perpetuity. It is made available under aCC-BY 4.0 International license.

from the analysis of ABE8e and amplicon sequencing. A) Predictions for results of ABE8e editing using marking by oligonucleotide incorporation at dsDNA breaks (iGUIDE). “Computational” denotes the prediction of incorporation by the nine bioinformatic panels. “ABE8e” denotes comparison to the rare oligonucleotide incorporations detected in the presence of the ABE8e editor. “ABE8e-Cas9^{WT}“ indicates comparison to sites of oligonucleotide incorporation in the presence of the ABE8e-Cas9^{WT} enzyme. “Cas9” denotes sites of oligonucleotide incorporation in the presence of the Cas9 editor. “No editor” indicates comparison to samples with no editor and so incorporation at background dsDNA breaks. B) Calls for sites of base editing compared to results from ABE8e and amplicon sequencing. “Computational” denotes pooled results of analysis from the two bioinformatic pipelines. “ABE8e-Cas9^{WT}“ denotes analysis of editing at sites of oligonucleotide incorporation directed by the ABE8e-Cas9^{WT} enzyme.

Supplementary Figure 4. Comparison of bases edited at off target sites.

A-I. Locations of edited bases at all off-target sites. The sequence of the matching. Samples are shown in the rows. A) Sites of base editing by the ABE8e-Cas9^{WT} at sites of oligonucleotide incorporation. B) Sites of base editing generated by the ABE8e base editing analyzed using the amplicon panel. C) Sites of base editing predicted by two bioinformatic tools. The extent of base editing is color-coded as indicated at the bottom. Numbering of the protospacer site starts on the left with “0”. The Cas9-targeted cut site is marked with the

bioRxiv preprint doi: https://doi.org/10.1101/2025.08.26.667396; this version posted August 26, 2025. The copyright holder for this preprint (which was not certified by peer review) is the author/funder, who has granted bioRxiv a license to display the preprint in perpetuity. It is made available under aCC-BY 4.0 International license.

vertical dash. Asterisks and tildes indicate the site is in a transcription unit and in an oncogene, respectively.

Figure S1

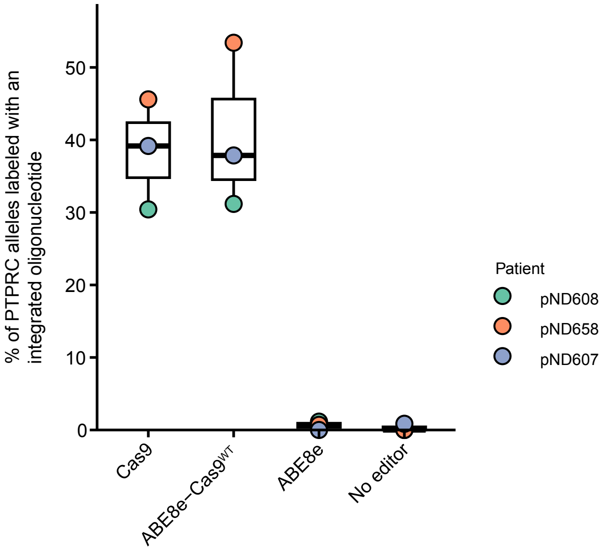

% of PTPRC alleles labeled with an integrated oligonucleotide
0
10
20
30
40
50
Cas9
ABE8e–Cas9WT
ABE8e
No editor
Patient
pND608
pND658
pND607

Figure S2

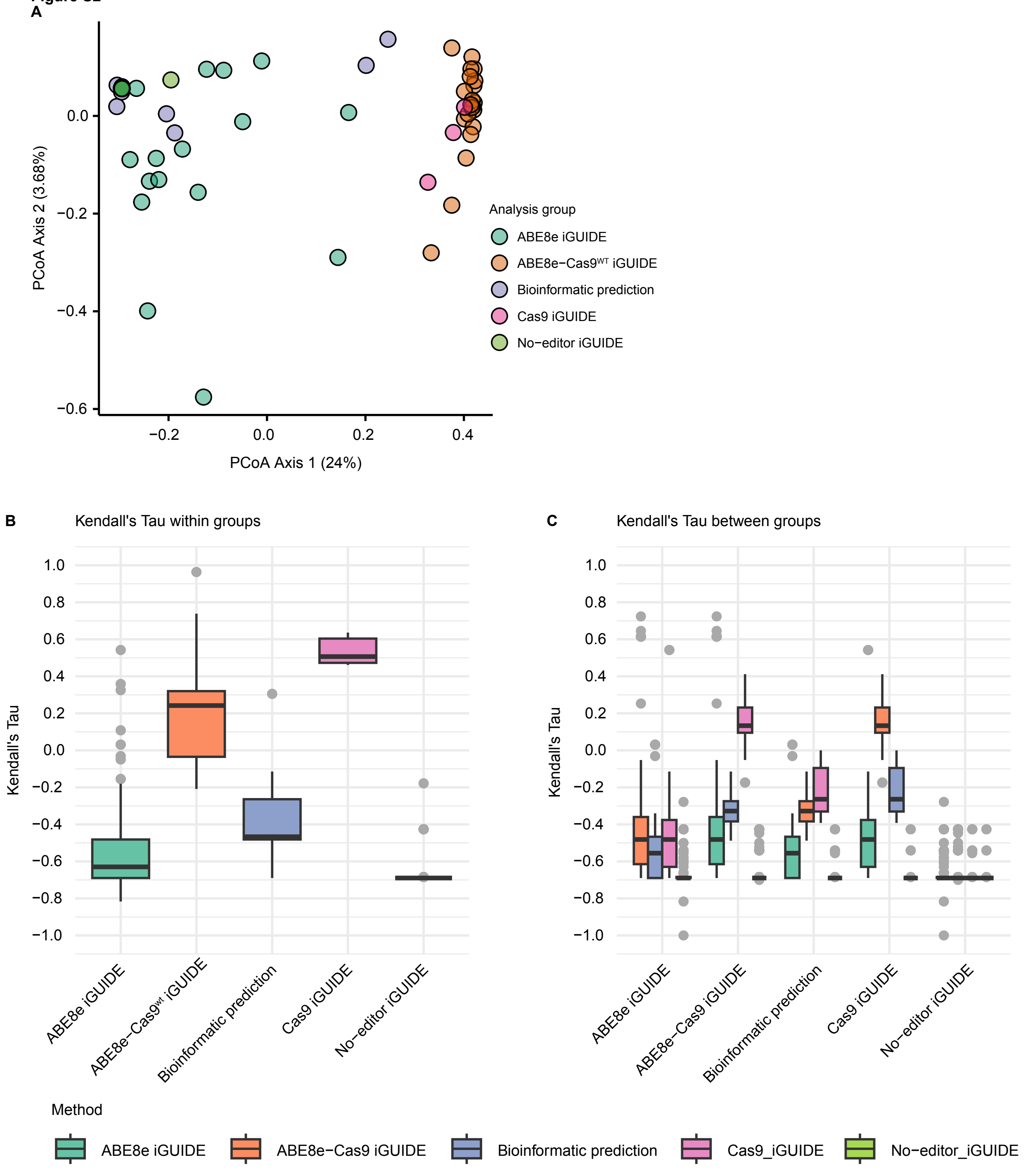
A
PCoA Axis 2 (3.68%)
PCoA Axis 1 (24%)
0.0
−0.2
−0.4
−0.6
−0.2
0.0
0.2
0.4
Analysis group
ABE8e iGUIDE
ABE8e−Cas9WT iGUIDE
Bioinformatic prediction
Cas9 iGUIDE
No−editor iGUIDE
B
Kendall's Tau within groups
Kendall's Tau
1.0
0.8
0.6
0.4
0.2
0.0
−0.2
−0.4
−0.6
−0.8
−1.0
ABE8e iGUIDE
ABE8e−Cas9Wt iGUIDE
Bioinformatic prediction
Cas9 iGUIDE
No−editor iGUIDE
C
Kendall's Tau between groups
Kendall's Tau
1.0
0.8
0.6
0.4
0.2
0.0
−0.2
−0.4
−0.6
−0.8
−1.0
ABE8e iGUIDE
ABE8e−Cas9 iGUIDE
Bioinformatic prediction
Cas9 iGUIDE
No−editor iGUIDE
Method
ABE8e iGUIDE
ABE8e−Cas9 iGUIDE
Bioinformatic prediction
Cas9_iGUIDE
No−editor_iGUIDE

Figure S3

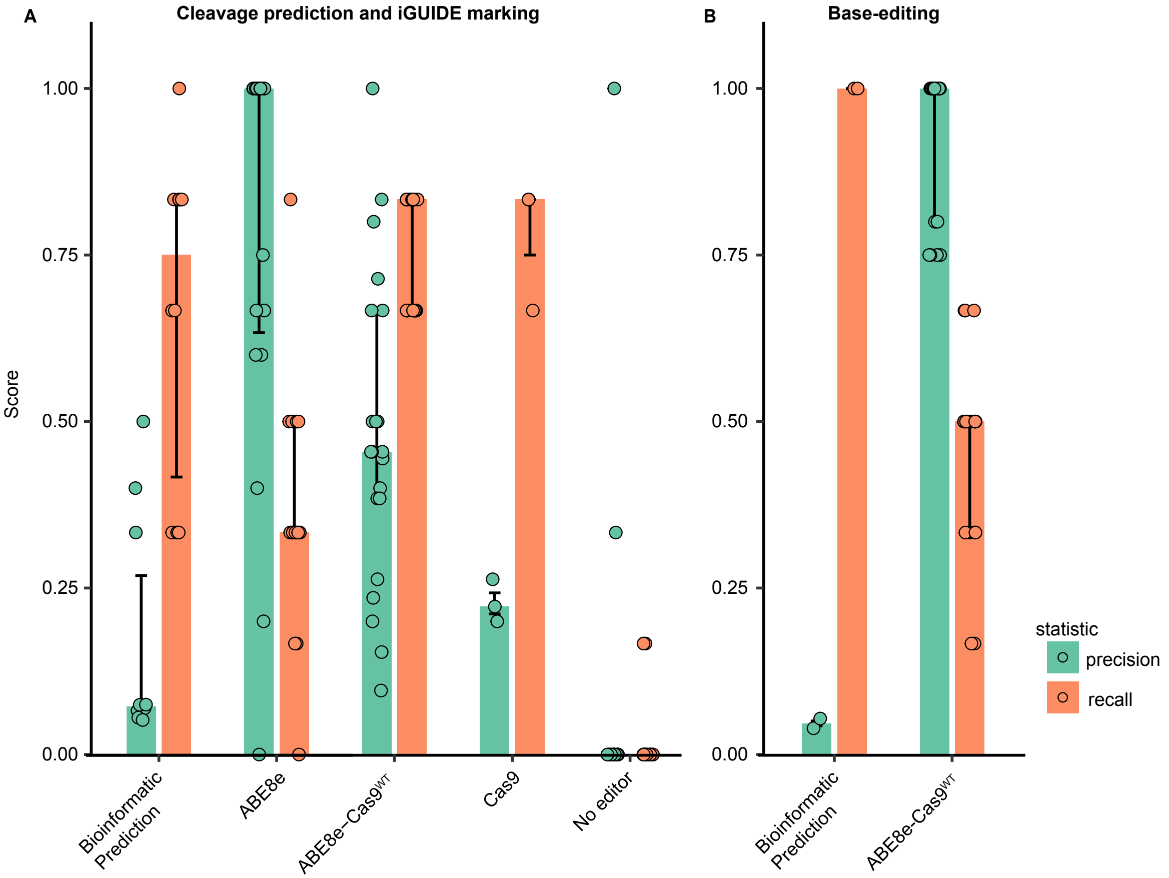
A
Cleavage prediction and iGUIDE marking
B
Base-editing
Score
1.00
0.75
0.50
0.25
0.00
Bioinformatic Prediction
ABE8e
ABE8e–Cas9WT
Cas9
No editor
statistic
precision
recall

Figure S4

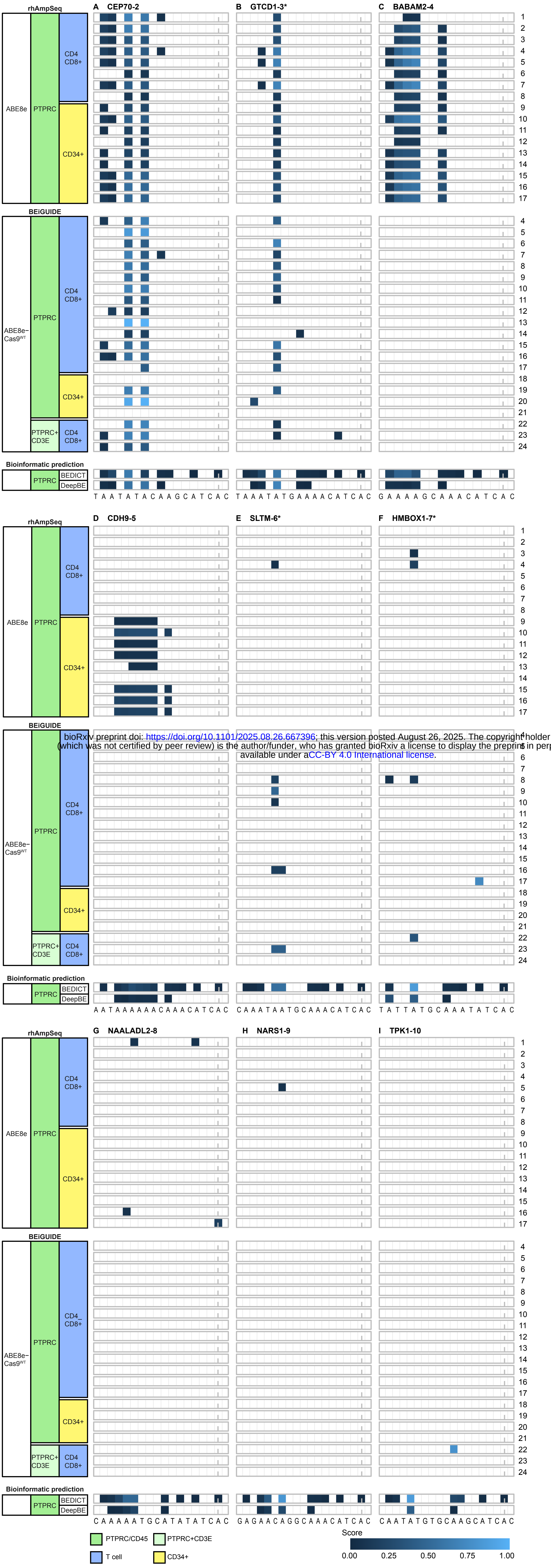

bioRxiv preprint doi: https://doi.org/10.1101/2025.08.26.667396; this version posted August 26, 2025. The copyright holder [illegible] (which was not certified by peer review) is the author/funder, who has granted bioRxiv a license to display the preprint in perp[illegible] available under aCC-BY 4.0 International license.
